# Fungal β-1,3-glucans: Cell Wall Constituents That Promote Gut Health Through Innate Immune Modulation

**DOI:** 10.3390/nu18111794

**Published:** 2026-06-02

**Authors:** Fnu Samiksha, Drishtant Singh, Sudi Shatha Harbool, Luca Di Martino, Caroline Kruithoff, Thomas S. McCormick, Mahmoud Ghannoum

**Affiliations:** 1 Department of Dermatology, Case Western Reserve University, Cleveland, OH 44106, USA; fxs313@case.edu (F.S.); ssh127@case.edu (S.S.H.);; 2 Department of Nutrition, Case Western Reserve University, Cleveland, OH 44106, USA; 3 Digestive Health Research Institute, Case Western Reserve University, Cleveland, OH 44106, USA; 4 Heritage College of Osteopathic Medicine, Ohio University, Cleveland OH 44122, USA; 5 Center for Medical Mycology, University Hospitals Cleveland Medical Center, Cleveland, OH 44145, USA

**Keywords:** β-1,3-glucan, immunomodulators, gut health, microbiome, anticancer

## Abstract

Fungal β-1,3-glucans are structurally conserved polysaccharide components of the fungal cell wall that exhibit potent immunomodulatory activity. These molecules are recognized by pattern recognition receptors, Toll-like receptors, complement receptor 3, lactosylceramide, scavenger receptors, and EphA2. Binding of β-1,3-glucans through these receptors triggers coordinated innate and adaptive immune responses such as cytokine production, phagocytosis, and trained immunity. In addition to receptor-mediated immune activation, dietary β-1,3-glucans function as fermentable prebiotic fibers that modulate gut microbiota composition, increase short-chain fatty acid production, and strengthen epithelial barrier integrity. These combined immunological and microbiome-mediated effects position β-1,3-glucans as key regulators of gut homeostasis. Preclinical and emerging clinical evidence supports broad therapeutic potential across multiple disease domains, including inflammatory bowel disease, metabolic disorders, respiratory infections, and cancer. In oncology, β-1,3-glucans enhance anti-tumor immunity, improve responses to monoclonal antibodies and chemotherapy, and serve as promising adjuvants in vaccine-based strategies. Additionally, β-1,3-glucan is widely used as a biomarker for invasive fungal infections and represents a validated target of antifungal therapies such as echinocandins. Despite these advances, clinical translation remains limited by heterogeneity in glucan source, structure, and formulation, as well as a lack of appropriately powered, standardized human clinical trials. Future efforts should focus on clarifying mechanisms of action, as well as rigorous clinical evaluation, to fully define the therapeutic utility of fungal β-1,3-glucans.

## 1. Introduction

Glucans are polysaccharides composed entirely of glucose monomers and are generally classified into three structural categories: α-glucans, β-glucans, and mixed α, β-glucans [1,2]. Examples include cellulose, glycogen, lentinan, and dextrose. Their classification and biological properties are primarily determined by molecular mass and the configuration of their glycosidic linkages [2]. Different types of glucans are found across diverse organisms.

β-1,3-glucans are naturally occurring polysaccharides present in bacteria, plants, and fungi. In fungi, they serve essential structural roles within the cell wall and are required for viability. Because β-1,3-glucans are absent in mammals, they represent attractive physiologically active targets for antifungal chemotherapies [3,4,5]. Fungal β-1,3-glucans play critical roles in inflammatory, infectious, and metabolic disorders, as well as in cancer immunity [6,7,8].

In addition to their roles in disease biology, β-1,3-glucans have demonstrated therapeutic potential as immunomodulators, vaccine adjuvants, and adjuncts in cancer therapy, with multiple formulations entering clinical use [9,10]. Recent advances have highlighted the significant impact of fungal cell wall β-1,3-glucans on gut health. By interacting with innate pattern recognition receptors (PRRs), including Dectin-1 and Toll-like receptors (TLRs), expressed on gut-associated lymphoid tissue (GALT) and immune cells, β-1,3-glucans regulate both innate and adaptive immune responses [6,11]. Moreover, they function as fermentable prebiotic fibers in the lower intestine, promoting beneficial microbial growth and strengthening the gut barrier [6,11,12,13]. This review provides a framework for understanding the translational significance of fungal β-1,3-glucans by examining their structure, sources, biological functions, roles in disease and cancer, and clinical applications.

## 2. The Fungal Cell Wall as a Source of Dietary β-glucans

### 2.1. Structural Characteristics of Fungal β-1,3-glucans

**Figure 1:**
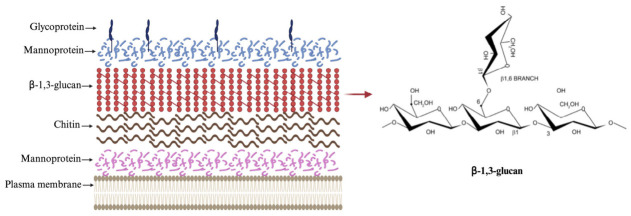
Components of fungal cell wall. Created in https://BioRender.com.

β-1,3-glucan is a major constituent of all characterized fungal cell walls (Figure 1), accounting for approximately 30% to 80% of cell wall mass [14]. In the fungal cell wall, β-1,3-glucans are typically ~1500 glucose units in length and consist of linear backbones of β-1,3-linked D-glucopyranosyl units, with side chains of D-glucopyranosyl residues attached via β-1,6 linkages [14,15,16,17,18].

In a study by Tada et al. [19], the structure of grifolan-LE, isolated from the mushroom *Grifola frondosa*, was characterized as a β-(1→3,1→6)-D-glucan consisting of a β-(1→3)-linked backbone with single β-(1→6)-linked side chains attached approximately every three backbone residues. In another study, Lowman et al. [18] used high-field NMR to characterize β-(1→6)-linked side chains in *Candida glabrata*, demonstrating average side chain lengths of 4–5 residues spaced approximately every 21 units along the β-(1→3) backbone.

Linear β-1,3-glucans with minimal branching can exist as single helical polymers or as right or left handed triple-helix structures [20]. This branching architecture facilitates its interaction with different receptors like Dectin-1 and TLRs on immune cells. This interaction triggers strong immune responses such as stimulating macrophages and natural killer (NK) cells, enhancing the body’s defense against infections, cancer, and inflammation [21,22].

Compared to cereal glucans, which have high solubility and high fermentability (easily fermented by gut microbiota producing short-chain fatty acids (SCFAs)), fungal β-glucans exhibit lower solubility and moderate fermentability [23]. Considering these differences is essential when evaluating the possible health impacts of β-glucans from various sources. The differences between the cereal and fungal **β-glucans** are presented in Table 1.

### 2.2. Dietary Sources

β-1,3-glucans occur in both prokaryotes (bacteria) and eukaryotes [fungi (yeast and molds)), and higher plants], where they function as structural or reserve polysaccharides (i.e., complex carbohydrates serving as long-term energy storage). They have been isolated from diverse sources, including cereals, mushrooms, and seaweed.

**Figure 2:**
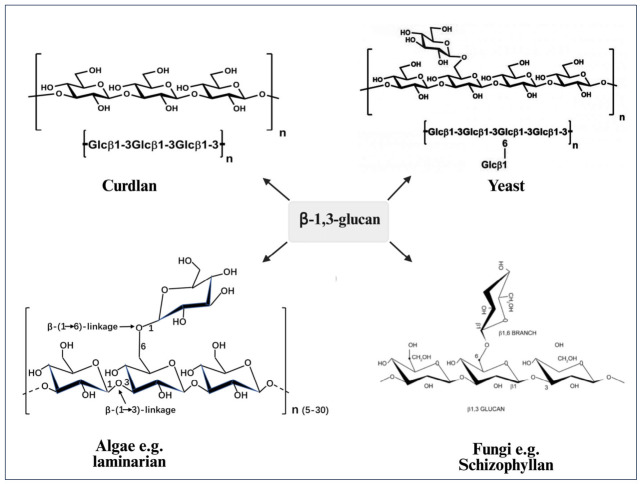
Primary structures of β-1,3-glucan from different sources. (Created in https://BioRender.com).

Naturally occurring β-1,3-glucans include curdlan (an exopolysaccharide from *Alcaligenes faecalis* composed of an unbranched β-D-glucan backbone), pachyman (similar to curdlan but with a lower degree of polymerization and occasional β-1,6 branching), paramylon (an intracellular storage polysaccharide found in certain protozoa such as euglenids), and scleroglucan (a fungal polysaccharide produced by *Sclerotium* species, consisting of a β-1,3-linked backbone with single β-1,6-linked glucosyl side chains attached to every third residue) [24]. The primary structures of β-1,3-glucans from these sources are illustrated in Figure 2. Fungal β-glucans have strong immunomodulatory properties, which makes them an excellent source for developing nutritional supplements and therapeutics that can boost immune system function [25]. Yeasts such as *Saccharomyces cerevisiae* and *Pichia* and medicinal mushrooms like *Lentinula edodes* and *Ganoderma lucidum* are rich sources of β-glucans and have long been utilized in traditional medicine for their immune-boosting properties.

A summary of β-glucans derived from different fungal sources and their associated therapeutic properties is provided in Table 2.

## 3. Immunomodulatory Effects of Fungal β-1,3-glucan

Fungal β-glucans regulate both innate and adaptive immune systems by directly interacting with immune cells, which boosts the body’s defensive responses by enhancing pathogen clearance and tissue repair [11]. β-glucans activate specific receptors on immune cells such as macrophages, neutrophils, and dendritic cells. They also modulate adaptive immunity, including T and B cell responses [64].

The initiation of immune responses to fungal β-glucan begins with its recognition by PRRs on immune cell surfaces. Although multiple receptor systems are involved, the overall framework is conserved and relies on the detection of pathogen-associated molecular patterns (PAMPs) by PRR families, including Dectin-1, TLRs, complement receptor 3 (CR3), lactosylceramide (LacCer), and scavenger receptors (SRs). These receptor systems and their downstream signaling pathways are discussed below.

**Figure 3:**
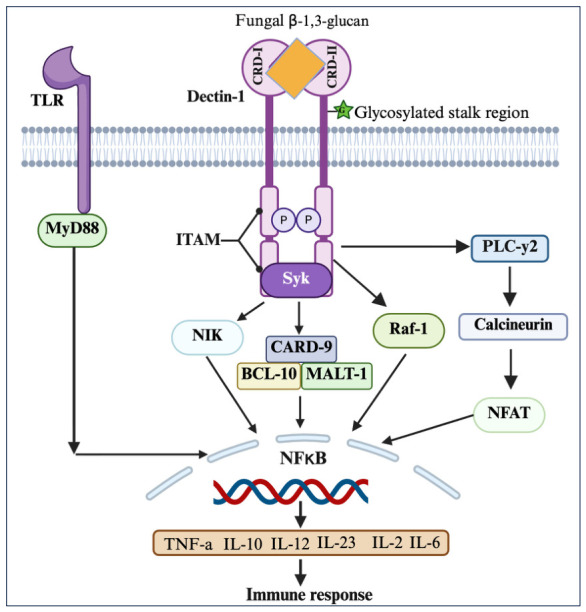
Schematic representation of downstream signaling induced by the interaction of β-glucan with the Dectin-1 receptor. Sensing of fungal β-glucan by Dectin-1 activates syk-dependent and -independent pathways. Downstream signals from Syk dependent pathways lead to activation of CARD9-Bcl10-Malt-1 and NIK signaling, whereas downstream signals from Syk-independent pathways lead to activation of Raf-1 and PLC-γ2 signaling which in turns activates NFкB and subsequent expression of different cytokines, like TNF-alpha, IL-1, IL-10, IL-12 and IL-6. CRD: carbohydrate recognition domain. Created in https://BioRender.com.

Dectin-1 is expressed on macrophages, T cells, neutrophils, and dendritic cells. Upon binding β-1,3-glucan, it triggers phagocytosis and release of cytokines (TNF, IL-6, IL-1, and CXCL8), initiating an inflammatory response for pathogen elimination [65,66]. In addition, it also initiates intracellular signaling pathways that are both TLR-dependent and TLR-independent [67]. See Figure 3. 

The Dectin-1– β -glucan axis is crucial for host defense but also regulates the response of the mucosal immune system to fungal products, thereby contributing to intestinal homeostasis [21,68]. Different studies also suggested that fungal β-glucans may alter the composition of colonic microbes and the immune response [6,23].

During fungal infection, Dectin-1 frequently cooperates with TLRs, particularly TLR2 and TLR4, to amplify inflammatory responses [69]. TLR signaling occurs primarily through the MyD88-Mal adaptor complex, activating NF-κB and MAP kinase pathways. Multiple studies have demonstrated convergence between Dectin-1 and TLR signaling pathways [69,70,71], enhancing cytokine production and promoting dendritic cell activation, Th17 differentiation, and cytotoxic T-cell responses [72,73,74,75].

TLR-independent Dectin-1 signaling is initiated upon β-glucan binding and phosphorylation of the hemITAM motif, which recruits spleen tyrosine kinase (Syk) and activates the CARD9-Bcl10-Malt1 complex, leading to NF-κB activation [75,76,77]. In addition to CARD9-dependent pathways, Dectin-1 activates CARD9-independent signaling, including ERK MAP kinase pathways, which regulate cytokines such as IL-2 and IL-10 [78,79]. NF-κB activity is also modulated by a Syk-independent Raf-1 pathway, which fine-tunes cytokine responses [80]. Additionally, Dectin-1 activates NFAT signaling in non-TLR contexts, regulating genes involved in immune responses, including COX-2, IL-2, IL-12p70, and IL-10 [81,82]. Beyond transcriptional control, Dectin-1 mediates non-opsonic phagocytosis through ITAM-dependent pathways involving PI3K, Vav1, and Rho GTPases, which can occur independently of Syk, highlighting cell-type-specific signaling flexibility [83].

Different TLRs recognize distinct extracellular and intracellular components of the fungal cell. Fungal β-glucan can activate TLR2, leading to NF-κB signaling and TNF-alpha production, as well as modulation of antigen-presenting cell function and immune tolerance. These effects are primarily mediated through the adaptor protein MyD88 [84,85,86,87].

**Figure 4:**
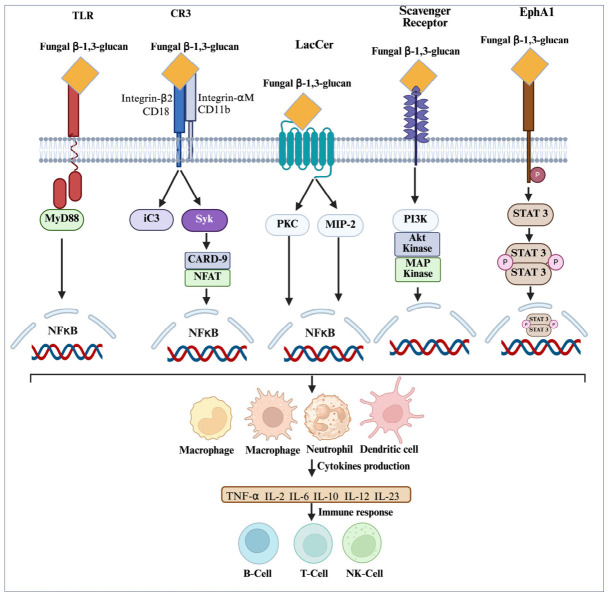
Toll-like receptors (TLRs), complement receptors (CR3), lactosylceramide (LacCer), scavenger receptors (SR), and EphA1 interacting with β-(1,3)-glucan and their downstream signaling pathways, resulting in immunological response. Created in https://BioRender.com.

CR3, also known as Mac-1, is a heterodimeric integrin composed of CD11b (αM) and CD18 (β2) subunits [88]. It is expressed on the surface of NK cells, neutrophils, and macrophages (Figure 4) [89]. Two distinct binding sites have been identified within CD11b: a C-terminal domain that binds β-glucans and an *N*-terminal domain that recognizes the complement fragment iC3b [90,91,92]. Binding of fungal β-glucan to CR3 enhances its affinity for iC3b, activating pathways that promote fungal and tumor cell cytotoxicity as well as phagocytosis [91,93]. Kunanopparat et al. [94] demonstrated that *Candida glabrata* β-glucan can activate regulatory T-cell signaling by modulating dendritic cell responses through CR3 and Dectin-1. Earlier studies showed that CR3 binds opsonized zymosan [95]. In murine models, Dectin-1 mediates β-glucan recognition, whereas CR3 is required for effective binding and responses to opsonized zymosan in humans [96].

LacCer is a neutral glycosphingolipid located on neutrophils and endothelial cells, where it forms membrane microdomains (Figure 4) [97]. LacCer directly binds fungal β-glucans through carbohydrate–carbohydrate interactions and activates Syk family kinases and PI3K signaling pathways. This activation induces downstream responses including NF-κB activation, chemotaxis, and cytokine secretion [98,99,100].

SRs are a heterogeneous class of PRR with broad ligand specificity, including microbial components, β-1,3-glucans, and lipoproteins, making them important contributors to antifungal immunity [101]. Different SR classes respond variably to fungal β-glucan exposure.

EphA2 is an epithelial cell receptor for β-glucans and belongs to the receptor tyrosine kinase (RTK) superfamily (Figure 4). Swidergall et al. [102,103] demonstrated that EphA2 functions as a neutrophil receptor for *Candida albicans* and promotes antifungal activity during oropharyngeal infection. Binding of *C. albicans* to EphA2 on oral epithelial cells activates STAT3 and MAP kinase signaling pathways, which are required for induction of proinflammatory and antifungal responses. Collectively, these findings highlight EphA2 as a multifunctional receptor; however, further studies are required to clarify its role in fungal infections and its broader relevance in disease and malignancy.

## 4. Functional Roles of Fungal β-glucans

As structural components of the fungal cell wall, dietary fungal β-glucans are increasingly recognized as important modulators of gut health. Beyond their direct receptor-mediated immune effects described in Section 3 above, these polysaccharides function as fermentable prebiotic fibers. Their structural diversity influences fermentation by the intestinal microbiome, selectively promoting the growth of beneficial bacteria such as *Lactobacillus* and *Bifidobacterium* [104,105] while suppressing opportunistic pathogens [104,106]. Through microbiome remodeling and enhancement of intestinal barrier integrity, β-1,3-glucans may provide protection against dysbiosis-associated inflammatory conditions.

### 4.1. Modulation of the Gut Microbiome

Fungal β-glucans have been reported as essential modulators of the gut microbiome. They can be utilized by specific intestinal microbes and could modify community composition in ways beneficial to the gut health of the host. Their structural diversity influences microbial utilization and downstream immune interactions. Through metabolite production and microbiome reshaping, β-glucans support intestinal barrier function and anti-inflammatory processes that may protect against dysbiosis related diseases like inflammatory bowel disease (IBD) including Crohn’s disease and ulcerative colitis, liver diseases, obesity, and cancer [13].

Yu and colleagues ([107] demonstrated that β-1,3-glucans can delay gastric emptying, while in the colon they promote growth of beneficial bacteria that regulate appetite-related hormones such as glucagon-like peptide-1 (GLP-1) and peptide YY (PYY). Another study [108] showed that chitin glucan (CG) from fungal sources improved glucose and lipid metabolism in high-fat diet induced obese mice, potentially through restoration of gut microbiota composition, particularly clostridial cluster XIVa. Qiao et al. [109] developed an enzymatically derived β-glucan from *S. cerevisiae* (BYG: Baker’s Yeast β-Glucan) using β-1,6-glucanase digestion and evaluated its effects in DSS-induced colitis. BYG modulated microbiota-derived SCFA production and reduced oxidative stress markers (NO, MDA, MPO), inflammatory mediators (NLRP3, ASC, caspase-1, iNOS, COX-2), and pro-inflammatory cytokines (IL-1β, IL-6, TNF-α, IFN-γ), while increasing expression of tight junction proteins (ZO-1, occludin, claudin-1).

Fungal β-glucan has been reported as a dietary polysaccharide that promotes the growth of specific gut-associated bacteria, such as *Bacteroides* and *Bifidobacterium*, which are considered helpful probiotic bacteria. Different studies have indicated that gut bacteria, particularly of genus *Bacteroides*, use a polysaccharide locus for the primary degradation of β-1,3-glucans [110,111]. The products formed following primary degradation (oligosaccharides) are then fermented by secondary degraders, including certain species of *Bifidobacterium* and *Lactiplantibacillus*, demonstrating microbial cross-feeding interactions [105]. This interaction favors a stronger interconnected microbial system and can contribute to the maintenance of a community framework linked to intestinal homeostasis [111]. Fungal β-glucans thus encourage the growth of good microbes, restoring microbial balance and alleviating dysbiosis-related diseases.

Collectively, fungal β-glucans influence the gut microbiome by enhancing specific microbial decomposition, facilitating cross-feeding interactions, and sustaining microbial populations associated with gut health. Their effects on fermentation and metabolite production indicate a potential pathway for barrier support, immune system balance, and overall gut homeostasis.

### 4.2. Epithelial Interactions

The gastrointestinal epithelium is the primary barrier between the host and the luminal contents, which include commensal microorganisms, food antigens, and microbial-derived polysaccharides [112,113,114]. Fungal β-glucans contribute to epithelial integrity by maintaining tight junction proteins (ZO-1, occludin, claudin-1, and JAM-1), which keep neighboring epithelial cells intact and thus reducing intestinal permeability [115,116]. This role is crucial since it prevents any toxic products/metabolites from crossing the epithelial barrier and entering the bloodstream, which may trigger immune cells and initiate inflammatory cascades [117]. Orally administered yeast β-glucans can enhance epithelial barrier functions, as demonstrated in a study by Han et al. [116], where yeast β-glucan improved DSS-induced changes in mucosal inflammatory lesions and the intestinal barrier by inhibiting the expression of inflammatory mediators (iNOS, COX-2 and PEG2) TNF-α, IL-6, and IL-8) and enhancing the expression of tight junction proteins (ZO-1, occludin, claudin-1, and JAM-1) associated with intestinal permeability. 

Moreover, fungal β-glucans may improve epithelial resilience by altering the surrounding microbiota and enhancing the production of SCFA, particularly butyrate [118,119]. These compounds nourish colonocytes and aid in barrier repair mechanisms. As a result, β-glucan exposure may assist the epithelium in recovering from stress and resisting damage caused by dysbiosis, infection, or persistent inflammation [23,120]. This part is especially important in diseases where epithelial dysfunction starts early and can make inflammation worse. In such cases, barrier disruption is not only a result of disease but also a cause of increased immune activity and microbial imbalance [121,122]. As a result, fungal β-glucan’s ability to maintain epithelial stability could be a key mechanism linking gut microbiota alterations and improved intestinal immunity and immune homeostasis. This interaction supports a more cooperative microbial network and may help maintain a community structure associated with intestinal homeostasis.

### 4.3. Inflammation and Oxidative Markers

Numerous studies have reported that fungal β-glucans exhibit broad disease modifying effects through immunomodulatory, antioxidant, and anti-inflammatory mechanisms. 

In a rat model of intra-abdominal sepsis caused by cecal ligation and puncture (CLP), β-1,3-glucan treatment reduced neutrophil influx, decreased myeloperoxidase activity, and lessened secondary lung injury and hemorrhage compared to untreated controls [123]. Similarly, β-1,3/1,6-glucan administration in a CLP-induced sepsis model preserved glutathione levels, reduced lipid peroxidation and myeloperoxidase activity, and normalized elevated TNF-α levels, indicating protection against systemic oxidative and inflammatory injury [124]. Although these findings are based on acute injury models, the underlying mechanisms are relevant to chronic disease states characterized by sustained oxidative stress and low-grade inflammation, suggesting broader therapeutic potential.

Pan et al. [125] showed that β-(1,3)/(1,6)-glucan from *L. edodes* stops cognitive decline in mice on a long-term high-fat diet by improving synaptic ultrastructure, reducing neuroinflammation, and restoring brain-derived neurotrophic factor (BDNF) levels. These effects were likely mediated through modulation of the gut-brain axis.

In contrast, an experimental study using *C. albicans* demonstrated that macrophages from patients with Crohn’s disease produce elevated levels of pro-inflammatory cytokines, including TNF-α, IL-6, and IL-1β, in response to β-1,3-glucan stimulation, thereby contributing to intestinal inflammation [126]. However, β-glucans have also been reported to improve insulin sensitivity, regulate glucose and lipid metabolism, and influence appetite control, thus supporting gut health [127]. 

Collectively, these findings support a role for β-1,3-glucans as immunomodulatory agents with potential applications across a broad spectrum of chronic inflammatory and metabolic diseases.

### 4.4. Systemic Effects

During invasive fungal infections (IFIs), β-1,3-glucan is released into the bloodstream, making it a clinically useful biomarker for fungal detection and diagnosis. In this regard, serum β-1,3-glucan (BDG) assays are widely used as rapid, non-culture-based screening tools for early identification of IFIs [128,129].

β-1,3-glucan levels may also serve as indicators of treatment response. In patients with invasive candidiasis treated with anidulafungin, declining serum β-1,3-glucan levels were associated with successful therapeutic outcomes [130]. These findings support the use of β-1,3-glucan as a biomarker to guide treatment monitoring and duration. β-1,3-glucan plays an active role in host immune responses to fungal infection. While recognition of β-1,3-glucan in superficial infections promotes effective fungal clearance, dysregulated responses in systemic infections can contribute to excessive inflammation, including cytokine storm-like syndromes. Kozłowska et al. [131] demonstrated that the β-glucans zymosan and curdlan increase expression of multiple PRRs, including Dectin-1, Dectin-2, TLR2, and TLR4, in human peripheral blood mononuclear cells. This was accompanied by increased reactive oxygen species (ROS), cytokines and chemokines production (IL17, IL22, IL23, IL6, TNF, CCL2, and TGF-β). Zymosan upregulated SOD1 expression, whereas curdlan reduced antioxidant enzymes including superoxide dismutase 1 (SOD1), catalase (CAT), and glutathione peroxidase 1 (GPX1), highlighting distinct effects on oxidative stress responses.

Torosantucci et al. [132] demonstrated that antibodies targeting β-1,3-glucan confer protection against both systemic and mucosal *C. albicans* infections by inhibiting key virulence factors such as hyphal formation and epithelial adherence.

### 4.5. Trained Immunity

Fungal β-glucan is a well-known activator of trained immunity when absorbed in the gut, which stimulates both systemic and mucosal host defense [133]. Orally administered fungal β-glucan interacts with the intestinal mucosa and is identified by PRRs on different immune cells like monocytes, neutrophils, and dendritic cells, activating downstream signaling pathways [21]. This interaction causes metabolic reprogramming along with long-term epigenetic changes. These alterations promote cytokine production, phagocytosis, and pathogen [134] death.

Fungal β-glucans promote trained immunity, providing long-term protection against secondary infections. Heterogeneity in β-glucan supply, structure, and solubility affects interaction with the phagocytic receptors, potentially impacting methods to increase trained immunity in humans [21]. As shown in a study by Pedro et al. [135] that Dectin-1-mediated recognition of β-glucans can induce epigenetic reprogramming (referred to as trained immunity), which results in enhanced host defense against subsequent infections.

Preclinical studies reveal that β-glucan-induced monocytes/macrophages improve vaccine effectiveness and provide better protection against microbial and viral exposures [136,137]. Furthermore, β-glucan has been shown to train peripheral monocytes, resulting in increases in cytokine responses and altered epigenetic and metabolic reprogramming over several weeks [134,138]. In terms of translational applications, trained immune responses could be utilized to improve innate immune function for therapeutic techniques such as vaccination or immunotherapy. This could also encourage improved resistance to novel infections for which we have no prior acquired memory [139,140].

### 4.6. Fungal β-Glucan and Gastrointestinal Tumorigenesis

#### 4.6.1. Effect on Colorectal Cancer

Colorectal cancer, often termed a “silent” disease, can progress to advanced stages before clinical symptoms become apparent. Consequently, development of effective adjunctive therapies remains a critical priority, particularly for patients diagnosed at later stages who require aggressive treatment.

Kim et al. [141] investigated the effects of low-molecular-weight fungal β-1,3/1,6-glucan on colorectal cancer cells. Their findings demonstrated that this glucan induces apoptosis in CT-26 colon cancer cells through activation of caspases and disruption of mitochondrial membrane potential. In vivo, treatment significantly reduced tumor size without detectable toxicity, suggesting potential as a therapeutic adjunct with a favorable safety profile.

Lentinan, a β-1,3-glucan, is already used in Japan as an adjunct therapy for gastric cancer and has also been evaluated in colorectal cancer. Hazama et al. [142] reported that superfine dispersed lentinan (SDL) improved symptoms in patients with advanced colorectal cancer, particularly in those with lower baseline quality of life scores. SDL also enhanced quality of life in patients undergoing chemotherapy and demonstrated greater efficacy in individuals with higher monocyte binding capacity.

Earlier clinical studies by Wakui et al. [143] showed that combining lentinan with chemotherapy significantly prolonged survival compared to chemotherapy alone in patients with advanced colorectal cancer, supporting its role as an effective adjunctive therapy.

Additional studies by Yoon et al. [144] and Binmama and co-worker [145] demonstrated that β-glucan derived from *S. cerevisiae* activates macrophages and T cells in colorectal cancer models, resulting in reduced tumor volume and decreased recurrence following surgical intervention.

#### 4.6.2. Effect on Pancreatic Cancer

Pancreatic ductal adenocarcinoma (PDAC) remains one of the most aggressive malignancies, with limited effective therapeutic options and poor overall survival. Emerging evidence suggests that fungal β-1,3-glucans may provide adjunctive benefits by enhancing anti-tumor immune responses.

Martin et al. [146] conducted a Phase II clinical study evaluating oral β-glucan supplementation in patients with stage III PDAC undergoing irreversible electroporation (IRE), a technique that induces tumor cell death via high-voltage electrical pulses. Compared to patients receiving IRE alone, those receiving adjunctive β-glucan demonstrated improved outcomes, including a median disease-free interval of 18 months and a median overall survival of 32.5 months. Importantly, β-glucan supplementation was well tolerated, supporting its feasibility as an adjunctive therapy.

Geller et al. [147] further demonstrated the immunomodulatory effects of yeast-derived β-1,3-glucan in preclinical models of pancreatic cancer, a disease known for resistance to immunotherapy. Treatment induced recruitment and reprogramming of monocytes and macrophages via a CCR2-dependent pathway, enhancing their cytotoxic potential. These changes were associated with reduced tumor burden, prolonged survival, and improved responses when combined with standard immunotherapy.

β-1,3/1,6-glucans derived from black yeast have also shown clinical promise. Tsukada et al. [148] reported that Nichi BRITE β-glucan was safe and associated with reductions in pancreatic cancer biomarker CA19-9, as well as improved disease-free survival in patients with perioperative digestive tract cancers.

Collectively, these studies suggest that β-glucans may serve as safe and effective adjunctive agents in pancreatic cancer therapy, enhancing immune responses and improving clinical outcomes when combined with standard treatments.

#### 4.6.3. Effect on Gastric Cancer

Gastric cancer remains a highly lethal malignancy, with an estimated five-year survival rate of approximately 37.9%, underscoring the need for improved therapeutic strategies.

A Phase Ib trial by Chu et al. [149] evaluated a combination regimen consisting of yeast derived β-glucans, camrelizumab, and SOX chemotherapy in patients with advanced gastric adenocarcinoma. Approximately 60% of patients experienced tumor shrinkage, with a median overall survival of 14 months. Although 30% of patients developed grade ≥ 3 adverse events, most side effects were manageable. Treatment was also associated with enhanced immune responses, including increased IL-2, IFN-γ, and CD4+ T-cell levels. While this is promising, further studies are needed to confirm these findings.

Tanaka et al. [150] demonstrated that oral β-1,3-D-glucan with β-1,6 branching reduced pro-inflammatory cytokine levels and increased expression of protective factors such as HSP70 in models of gastric mucosal injury. Treatment also reduced neutrophil infiltration, suggesting a role in preserving gastric mucosal integrity. These findings are particularly relevant given that many gastric cancers originate from damage to the gastric mucosa.

Lentinan has been widely studied as an adjunct therapy in gastric cancer. In an early clinical study, Taguchi [151] reported that patients receiving lentinan in combination with tegafur chemotherapy demonstrated significantly improved survival compared to those receiving chemotherapy alone, with only mild and transient adverse effects. Similarly, Wakui et al. [143] reported improved survival outcomes in patients receiving lentinan in combination with chemotherapy, supporting its broader applicability across gastrointestinal malignancies.

However, results are not uniformly consistent. Higashi et al. [152] found no significant survival benefit in patients with unresectable gastric cancer treated with lentinan. Nevertheless, lentinan improved treatment tolerability, reduced chemotherapy-related adverse effects, and enhanced quality of life, allowing patients to continue therapy for longer durations.

#### 4.6.4. Effect on Esophageal Cancer

Esophageal cancer patients may also benefit from adjunctive β-glucan therapy, particularly with lentinan.

Del Cornò et al. [153] reported that patients receiving lentinan exhibited increased IL-12 and decreased IL-4 levels, indicating enhanced anti-tumor immune responses and reduced tumor-promoting signaling.

Similarly, Wang et al. [154] demonstrated that patients treated with a combination of lentinan and chemotherapy showed greater improvements in immune function compared to those receiving chemotherapy alone. Specifically, lentinan treatment increased levels of anti-tumor cytokines (IL-2, IL-6, and IL-12) while decreasing tumor-promoting cytokines (IL-4, IL-5, and IL-10).

In addition to immunological benefits, patients receiving lentinan reported improved quality of life and higher rates of remission, supporting its role as an effective adjunctive therapy in esophageal cancer.

## 5. Clinical Applications

### 5.1. Preclinical Applications

The role of fungal β-glucan has been demonstrated in many animal models, including pigs, canines, horses, calves, etc. Most of the animal models have demonstrated positive effects of fungal β-glucan on gut health by modulating the gut microbiome and immune responses. The following sections discuss some of the published animal models.

#### 5.1.1. Pigs

Fungal β-glucans have been reported to have positive effects on the gut health and growth performance of nursery pigs [155,156,157,158]. β-glucan from *S. cerevisiae* reduced the population of pathogenic bacteria (*Enterobacteria*) without influencing the *lactobacilli* and *bifidobacteria* populations in the ileum and colon of pigs, suggesting the potential role of yeast β-glucans in improving the gut health of nursery pigs [159]. The activation of the Dectin-1 receptor in the small intestine by β-glucan, which results in immune stimulation and the release of proinflammatory cytokines like TNF-α, IL-6, and IL-1 to activate macrophages for defense against infection, could be the probable cause of the improvement in intestinal health in nursery pigs [160,161].

#### 5.1.2. Dogs

In dogs with IBD, supplementation with β-glucan reduced Canine Inflammatory Bowel Disease Activity Index (CIBDAI) values, increased anti-inflammatory cytokine interleukin (IL)-10, and improved histopathological parameters [162]. Amaral et al. [163] investigated the effects of orally supplemented β-glucan and mannan-oligosaccharides on fecal microbiota and SCFA concentrations in dogs with moderate IBD and found that it positively altered the bacterial population of *Firmicutes* and *Bacteroidetes*, demonstrating favorable effects. In another study by de Souza Theodoro et al. [164] β-glucan (from *S*. *cerevisiae)* supplementation in dogs increased the serum levels of IL-2 and neutrophil phagocytic index, thus modulating the immune system and inflammatory activity.

#### 5.1.3. Horses

Lacerenza et al. [165] studied the effects of β-glucan supplementation on LPS-induced endotoxemia in horses and reported that it modulated the immune response by increasing serum total proteins, globulins, and IL-8 and changing the type of peritoneal inflammatory cells, without effectively attenuating clinical signs of endotoxemia in horses.

Picetti et al. [166] studied the effects of β-glucan (from yeast) as a food additive on blood leukocytes and selected innate immune parameters in English thoroughbred horses under regular training conditions. The study found that it enhanced complement-mediated immune activity and monocyte responses, making it a potential supportive immune supplement for horses before stressful events. 

#### 5.1.4. Broiler Chicken

Fungal/yeast β-glucans have been utilized in broiler chicken feeds for decades, and they have shown an increase in animal productivity due to their physiological effects on the intestinal digestive mucosa [167,168].

Other studies by Zhen et al. [169,170] indicated that yeast β-glucan induced significant escalation of systemic immunity and reduction in the mortality rate of laying hens and improved egg quality and fertile egg hatchability, suggesting beneficial effects of yeast β-glucan addition on the reproductive performance of aged hens. Yeast β-glucan was fed to breeder hens and modulated the gut microbiome (by enhancing beneficial microbes and reducing pathogenic bacteria) and microbial metabolite profiles. This study revealed a promising strategy for the prevention of age-related immune hypofunction or chronic intestinal inflammation in aged hens with the help of dietary supplement-based immunomodulators. 

#### 5.1.5. Calves

Wang et al. [171] studied the effects of yeast β-glucan supplementation on calf intestinal and respiratory health and demonstrated that feeding with yeast β-glucan twice effectively trains calves to cope with later stress, significantly reduces the incidence of diarrhea and pneumonia, and improves intestinal health. These benefits lead to the induction of trained immunity in the calves.

In another study by Yan et al. [172], it was demonstrated that intraperitoneal injection of yeast derived β-glucan to suckling Holstein dairy calves effectively reduced the frequency of diarrhea and bovine respiratory disease and improved the intestinal health status, which also suggested the involvement of trained immunity. 

#### 5.1.6. Zebrafish

A study by Liang et al. [173] showed that the dietary supplementation of yeast β-glucan enhanced the antiviral ability of zebrafish. Yeast β-glucan stimulated type-I IFN signaling in both adult and larval zebrafish after spring viremia of carp virus infection. Moreover, β-glucan altered the intestinal microbiota of zebrafish, which contributed to the antiviral function. 

#### 5.1.7. Models of Colitis and IBD

The protective effects of yeast derived β-glucan in IBD have been demonstrated in multiple studies. Li et al. [174] showed protective effects of yeast β-glucan in a DSS-induced colitis mouse model. β-glucan treatment significantly reduced disease severity, as evidenced by decreased weight loss, lower disease activity index scores, and reduced intestinal damage. These effects were associated with enhanced barrier integrity via upregulation of tight junction proteins, suppression of LPS release, and reduced pro-inflammatory cytokine production.

Han et al. [116] further demonstrated that yeast β-glucan reduces clinical symptoms, inflammatory cell infiltration, and epithelial apoptosis in dextran sulfate sodium (DSS)-induced colitis in C57BL/6 mice. Treatment improved intestinal permeability and preserved tight junction integrity, while also modulating immunoglobulin levels. These findings indicate that β-glucan ameliorates mucosal inflammation and barrier dysfunction by suppressing inflammatory mediators and enhancing tight junction protein expression.

Zhu et al. [175] evaluated the effect of crude β-glucan extracts from *Pichia kudriavzevii* DPUL-51–6Y, *Kluyveromyces marxianus* DPUL-F15, and *S. cerevisiae* DPUL-C6 on BALB/c Mice. These extracts significantly mitigated inflammatory responses by reducing lipopolysaccharide (LPS) induced nitric oxide production and pro-inflammatory cytokine release through suppression of NF-κB signaling. Additionally, they alleviated ulcerative colitis by reshaping the gut microbiota, increasing the abundance of Lactobacillus and Prevotella, and enhancing SCFA production in the intestinal tract.

Collectively, these studies demonstrated that yeast derived β-glucans exert protective effects in colitis and IBD models through coordinated modulation of gut microbiota, epithelial barrier integrity, and inflammatory signaling pathways.

Taken together, fungal β-glucans have shown tremendous potential in preliminary studies conducted using several animal models, providing a robust experimental foundation for defining their roles in gut health, microbiome modification, and systemic immunometabolic effects. However, human clinical trials are needed to confirm their effectiveness.

### 5.2. Therapeutic Use

Fungal β-1,3-glucan has gained increasing attention due to its potential therapeutic benefits. Numerous studies have demonstrated the biological activity of fungal β-glucans, and promising results from preclinical models have driven efforts to evaluate their efficacy in human populations (Table 3).

Early clinical trials indicate that fungal β-glucans are generally well tolerated in patients undergoing chemotherapy or radiation therapy, with minimal adverse effects reported [7,176,177,178,179,180]. Several studies have evaluated β-glucans as adjuncts to radiotherapy and chemotherapy, demonstrating improved recovery of peripheral blood mononuclear cells (PBMCs) and reduced treatment associated immunosuppression [181,182].

However, heterogeneity in study design, β-glucan source, dosing strategies, and clinical endpoints limits direct comparison across studies and underscores the need for larger, standardized clinical trials. Among the various β-glucans evaluated, lentinan is one of the most extensively studied in clinical oncology. When combined with chemotherapy in lung and gastric cancer, lentinan has been associated with improved treatment response and survival outcomes [33,183,184,185,186,187].

Other fungal β-glucans, including Imprime PGG, have been investigated in combination with immune checkpoint inhibitors and other therapeutic modalities [188,189,190,191].

### 5.3. Established Clinical Applications

#### 5.3.1. Safety

β-1,3-glucans represent a unique class of bioactive compounds capable of enhancing immune function and supporting host defense. They are widely used as dietary supplements and functional food ingredients. Historically, yeast derived glucans have even been recommended by organizations such as NASA as nutritional support to enhance immune resilience in astronauts. From a regulatory perspective, β-glucan derived from S. cerevisiae has been approved by the U.S. Food and Drug Administration (FDA) as a safe food additive. Regulatory agencies generally consider β-1,3-glucan to be safe when consumed at recommended doses, with few documented adverse effects. Oral yeast β-glucans have an acceptable safety and tolerability profile in healthy individuals and patients at regularly used doses of 100–500 mg/day [192,193].

#### 5.3.2. Modulating Gut Microbiome

The administration of EpiCor fermentate (a yeast fermentate made using *S. cerevisiae*) to 80 participants suffering from gastrointestinal discomfort and constipation altered the composition of the gut microbiome, resulting in an alleviation of constipation related symptoms [194]. In a clinical trial, supplementing 80 constipation prone individuals with β-glucan from *Schizophyllum* commune (TBG-136) improved gut health by increasing beneficial bacteria and improving bowel movement frequency and transit time, and improving their overall quality of life [195]. Pallav et al. [196] investigated the effect of polysaccharopeptide from *Trametes versicolor* on the gut microbiome of healthy volunteers and discovered that healthy persons’ microbiomes exhibit significant diversity while remaining stable over time. *T. versicolor*’s polysaccharopeptide serves as a prebiotic, modulating the composition of the human gut microbiome. In another clinical trial Ranaico et al. [197] found that chitin-glucan supplementation altered gut microbiota composition and improved postprandial glycemic response in 15 cardiometabolic risk subjects.

#### 5.3.3. Trained Immunity

A clinical trial (NCT03080974) investigated whether irreversible electroporation (IRE) can stimulate trained immunity in patients with pancreatic adenocarcinoma induced by yeast derived particulate *β*-glucan and showed that the combination of yeast-derived particulate *β*-glucan with irreversible electroporation ablated pancreatic adenocarcinoma tumor cells, elicited a strong trained response, and increased anti-tumor functionality 12 months after IRE, leading to improved disease free interval and overall survival [198].

#### 5.3.4. Stimulate Protective Immunity

A study by Auinger and co-workers demonstrated that the preparation of yeast (1→3)-β-glucan increased the body’s ability to protect against invading pathogens [199]. Meanwhile, another study by Carpenter et al. [200] demonstrated that fungal β-glucan has the potential to stimulate protective immunity without enhancing inflammation and modify immune responses following a strenuous exercise session. Finally, Medeiros and associates [201] investigated the impact of *S. cerevisiae* β-1,3-glucan on venous ulcer healing in humans and showed that it could serve as a natural biological response modifier for wound healing.

#### 5.3.5. Upper Respiratory Tract Infections

Daily consumption of yeast β-glucan provide protection against upper respiratory tract infections (URTIs). It also shortens the duration of URTI symptoms in infected older adults through boosting innate defenses by stimulating NK cell activity and phagocytosis [28,202,203,204]. Although these findings are encouraging there is a need to standardize β-glucan formulations and dosing use.

Overall, fungal β-glucans have favorable clinical evidence for respiratory infections [203]; immunological responses [199,205,206,207], allergies, and cancer supportive care. However, their ability to modulate the gut microbiome indicates potential benefits for gastrointestinal-related ailments. Clinical studies emphasizing gut outcomes, such as IBS and dysbiosis associated disorders, are scarce and a critical area for future research.

**Table 3:** Clinical trials conducted using fungal β-glucan.

Study	Study D1.	NCT	Participants	Source of (1→3)-β-Glucan	Dose & Duration	Clinical Indication/Disease	Primary outcome	Safety	Ref
β-D-glucan (BDG) surveillance with preemptive anidulafungin vs. standard care for invasive Candidiasis in surgical intensive care unit (SICU) patients (2008–2010)	Randomized, open-label, pilot clinical trial	NCT00672841	64 critically ill adult ICU patients hospitalized ≥ 3 days and at risk for invasive candidiasis	Circulating serum BDG measured using Fungitell™ assay	Twice-weekly BDG surveillance, preemptive anidulafungin initiated after ≥2 sequential BDG levels ≥ 80 pg/mL	Early detection and preemptive management of invasive candidiasis in at-risk ICU patients	Feasibility of BDG-guided preemptive antifungal therapy, ≥2 BDG levels ≥ 80 pg/mL demonstrated 100% sensitivity and 75% specificity for invasive candidiasis	Preemptive anidulafungin was safe and generally well tolerated	[208]
Discontinuation of empirical antifungal therapy in ICU patients using 1,3-β-D-glucan (2010–2014)	Prospective, randomized, controlled, open-label clinical trial	NCT01734525	Critically ill adult ICU patients with suspected invasive candidiasis receiving empirical antifungal therapy	Circulating serum BDG measured serially using Fungitell^®^ assay	Empirical antifungal therapy discontinued if BDG negative, BDG measured twice weekly during ICU stay	Suspected invasive candidiasis in critically ill ICU patients	BDG-guided strategy significantly reduced duration of empirical antifungal therapy without increasing incidence of invasive candidiasis or mortality	No significant differences in ICU mortality or development of invasive candidiasis between BDG-guided and standard-of-care groups	[209]
β-glucan driven vs. empirical antifungal therapy in critically Ill patients (2017–2019)	Single-center, open-label, randomized controlled clinical trial	NCT03117439	108 critically ill adult ICU patients with sepsis and risk factors for invasive candidiasis receiving empirical antifungal therapy	Circulating serum β-1,3-D-glucan measured serially (enrollment and every 48–72 h for up to 14 days)	Antifungal therapy discontinued if BDG negative, BDG monitored at enrollment and every 48–72 h over 14 days	Suspected invasive *Candida infection* in critically ill septic ICU patients	BDG-guided strategy significantly reduced duration of empirical antifungal therapy within 30 days compared with standard care	No significant difference in 30-day mortality or incidence of invasive candidiasis between BDG-guided and control groups	[210]
CandiSep Trial (2016–2019)	Open-label, randomized, multicenter clinical trial	NCT02734550	339 adult ICU patients with sepsis or septic shock at high risk for invasive Candida infection (ICI)	Circulating serum (β-1,3-D-glucan measured using Fungitell^®^ assay (Limulus amebocyte lysate-based)	Diagnostic threshold ≥ 80 pg/mL, two measurements within 24 h	Early diagnosis and management of invasive *Candida* infection in sepsis	BDG-guided antifungal therapy did not reduce 28-day all-cause mortality compared with standard care, resulted in earlier and more frequent antifungal use	No major safety concerns, increased antifungal exposure without mortality benefit	[211]
Irreversible electroporation and β-glucan induced trained innate immunity for treatment of pancreatic ductal adenocarcinoma	Prospective Phase II trial (IRE + adjuvant oral β-glucan vs. IRE alone comparator cohort)	NCT03080974	50 total (30 IRE + β-glucan, 20 IRE alone), Stage III locally advanced PDA	Yeast derived particulate β-glucan (Wellmune^®^, from **S. cerevisiae**)	500 mg orally twice daily (1000 mg/day) for 12 months or until progression	Stage III locally advanced pancreatic ductal adenocarcinoma	Median DFI of 18 months, median OS of 32.5 months, improved CD4/CD8 terminal effector phenotype correlating with survival	No dose-limiting toxicities, 96% compliance, 23% grade 3–4 AEs at 90 days (none required β-glucan dose modification), no 90-day mortality	[146]
Clinical trials of yeast-derived β-1,3-glucan in children: effects on innate immunity	Randomized, double-blind, placebo-controlled trial	Not reported	56 children (originally 60 enrolled, 4 excluded), aged approximately 8–12 years, with chronic respiratory problems, placebo group *(n = 27)* and glucan group *(n = 29)*	Yeast-derived insoluble β-1,3-glucan (#300), >85% purity (Transfer Point, Columbia, SC, USA)	100 mg/day orally for 30 days	Children with chronic respiratory problems, evaluation of nonspecific (innate) mucosal immunity	Changes in salivary markers of innate immunity and inflammation (albumin, lysozyme, C-reactive protein [CRP], calprotectin), significant reduction in albumin and calprotectin levels, significant modulation of lysozyme, CRP changes not statistically significant	No adverse effects reported, treatment was well tolerated under medical supervision	[212]
Randomized, double-blind pilot study examining yeast β-glucan supplementation on antibody titer response following influenza vaccination in older adults	Randomized, double-blind, placebo-controlled pilot trial (1:1 allocation)	Not reported	78 randomized participants (β-glucan *n = 38*, placebo *n = 40)*, adults aged 50–89 years	Yeast-derived insoluble β-1,3/1,6-glucan (manufacturer: Lallemand Inc.)	500 mg/day orally, supplementation included a 2-week pre-vaccination period and continued until 28 days post-vaccination	Immune response to seasonal influenza vaccination in generally healthy older adults (>50 years)	Mean change in Influenza A hemagglutination inhibition (HI) antibody titer from baseline to 28 days post-vaccination, β-glucan group showed significantly higher Influenza A antibody titer compared to placebo in Season 1 (*p* = 0.037)	No unexpected adverse events reported, few isolated fever cases (1 during β-glucan intervention, 1 during placebo, 1 at baseline), no differences in fatigue, cold/flu symptoms, or serious adverse events between groups	[207]
Effects of low-to-moderate dose yeast β-glucan (YBG) supplementation on upper respiratory symptoms and mood in moderately stressed adults	12-week randomized, double-blind, placebo-controlled trial	ISRCTN 48336189	198 moderately stressed adults (PSS-10: 14–26), 190 completed (Placebo *n = 65*, 120 mg *n = 62,* 204 mg *n = 63)*, mean BMI 24.2 ± 5.5 kg/m^2^	Yeast-derived β-(1,3/1,6)-glucan from *S. cerevisiae*	120 mg/day or 204 mg/day orally for 12 weeks	Upper respiratory tract infection (URTI) symptom severity in moderately stressed adults, mood and fatigue assessment	Change in WURSS-21 total severity score at week 12, both doses significantly reduced URTI severity vs. placebo (*p* = 0.002 and *p* < 0.001), only 204 mg exceeded minimal clinically important difference (MCID = 6.5), mood states significantly improved (*p* < 0.001)	No serious adverse events, no treatment related adverse events, biochemical markers remained within normal ranges, minor transient symptoms (GI discomfort, headache) not treatment-related	[213]
Effects of Yeast β-glucan Supplementation on Upper Respiratory Tract Infections in Older Adults (2013–2015)	Randomized, double-blind, placebo-controlled clinical trial	Not reported	Healthy adults aged 50–70 years *(n = 100)*	Yeast-derived β-1,3/1,6-glucan (Wellmune^®^, *S. cerevisiae*)	250 mg orally once daily for 90 days during winter months	Prevention of upper respiratory tract infections in older adults	Incidence and severity of URTI symptoms, β-glucan group showed reduced URTI symptom severity and improved immune response markers	Well tolerated, no significant adverse effects compared with placebo	[203]
Yeast (1,3)-(1,6)-β-glucan supplementation for immune defense in healthy adults (2009–2011)	Randomized, double-blind, placebo-controlled multicenter clinical trial	Not reported	162 healthy adults with recurrent infections	Insoluble yeast-derived β-(1,3)/(1,6)-D-glucan	900 mg/day orally for 16 weeks	Prevention of common cold infections / immune support	β-glucan supplementation reduced symptomatic common cold infections by ~25% vs. placebo	Well tolerated, no major safety concerns reported	[199]
Effects of orally administered β-glucan on innate immune responses in humans (2013)	Randomized open-label intervention pilot study	NCT01727895	15 healthy male volunteers	Oral commercially available water-insoluble β-glucan (yeast-derived)	1000 mg orally once daily for 7 days	Evaluation of β-glucan effects on innate immune responses in healthy humans	No significant effect on cytokine production or leukocyte microbicidal activity after supplementation	Well tolerated, no major adverse effects reported	[214]

## 6. Limitations, Controversies and Future Directions

This review highlights the significant therapeutic potential of fungal β-(1,3)-glucans across a range of conditions, including malignancies, IBD, and colitis. Numerous studies demonstrated that fungal β-glucans modulate immune responses through interactions with pattern recognition receptors such as Dectin-1, TLRs, CR3, LacCer, SRs, and EphA2.

Yeast derived β-glucans represent a major and accessible source of these compounds. Oral supplementation is generally well tolerated and has been associated with multiple health-promoting effects, particularly through immune system modulation.

However, several limitations remain. Human clinical data are relatively limited, and many studies have not been independently replicated.

The most common limitation among them is the product heterogeneity, as β-glucan purification differs by species, method used, extent of branching, etc. However, numerous investigations test non-equivalent products without providing detailed physicochemical characterization. Mechanistic inconsistency exists due to a variety of PRRs and receptor-mediated interaction contributing to context-specific signaling. Additionally, soluble and insoluble forms of β-glucan have different biologies which are often ignored. Moreover, some contaminants like mannans, endotoxin, etc., may interfere with immune response mediated by β-glucan. Some robust preclinical findings generally fail to reproduce in humans due to species differences in PRR biological activity, microbiota structure, and exposure protocols. The safety data of fungal β-glucan is also sparse in immunocompromised individuals.

Future research should focus on the development of standardized β-glucan preparations with well-defined biochemical and structural characteristics. Given that yeast culture systems are well established, scalable production of standardized β-glucans is feasible and may offer significant economic and biotechnological advantages.

Importantly, larger, well-controlled clinical trials are needed to validate the therapeutic efficacy of β-1,3-glucans and to define optimal dosing, formulation, and clinical indications.

## Figures and Tables

**Table 1:** Differences between cereal and fungal β-glucans.

Characteristics	Cereal β-glucans	Fungal β-glucans
Linkage	Mixed β-1,3 and β-1,4 linkages in a linear, unbranched chain	β-1,3 backbone with β-1,6 side branches
Branching	Unbranched	Branched
Molecular weight	Low to high	Variable
Conformation	Flexible chain conformations	Triple-helical or aggregated conformations
Solubility	More water-soluble; form viscous, high-molecular-weight solutions at physiological concentrations	High molecular weight, particulate/insoluble
PRR engagement and innate immunity	Minimal direct Dectin-1 activation; effects on innate immunity are generally indirect (via microbiota/SCFA or metabolic changes)	High affinity for Dectin-1 and CR3, cytokine production and trained immunity
Microbiota and metabolic effects	Fermentable which increases SCFAs	Less fermentable; chitin–glucan mixes can be fermented and modulate microbiota to a little extent
Vaccine/adjuvant and antigen carrier roles	Not used as classical particulate adjuvants	Effective antigen carriers/adjuvants
Translational implications	Cholesterol-lowering and prebiotic applications	Immune priming, vaccine adjuvancy and adjunctive immunotherapy applications

**Table 2:** List of β-glucan from different fungal sources with their therapeutic properties.

β-Glucan	Name of Fungus	Structure	Therapeutic Applications	Reference
Pleuran	*Pleurotus* spp.	Branched β-(1→3)/(1→6)	Immunomodulator, adjunct in respiratory infections, supportive anticancer therapy	[26,27,28,29,30]
Lentinan	*Lentinus edodes*	Branched β-(1→3)/(1→6)	Approved adjunct in gastric and colorectal cancer therapy, enhances chemotherapy response	[31,32,33,34]
Schizophyllan/Sizofiran/Sonifilan	*Schizophyllum commune*	Branched β-(1→3)/(1→6)	Adjuvant in cervical and head & neck cancers, immune stimulation	[35,36,37]
Gl–1	*Ganodema lucidum*	Branched β-(1→3)/(1→6)	Antitumor activity, immune enhancement, metabolic syndrome support	[38,39,40,41]
Krestin	*Trametes versicolor*	Branched β-(1→3)/(1→6)	Approved cancer adjuvant in Japan (gastric, colorectal, lung cancers), improves survival	[42,43,44,45,46]
Grifolan	*Grifola fondosa*	Branched β-(1→3)/(1→6)	Antitumor immunomodulator, macrophage and NK cell activation	[47,48,49,50]
Flammulin	*Flammulina velutipes*	Branched β-(1→3)/(1→6)	Anticancer and immunostimulatory effects, antioxidant activity	[51,52,53]
Pachymaran	*Poria cocos*	Linear β-(1→3)	Antitumor immunomodulator, enhances macrophage and NK cell activity, supportive therapy in cancer and inflammatory disorders	[54,55,56,57]
Maitake	*Grifola frondosa*	Branched β-(1→3)/(1→6)	Anticancer adjuvant, immune enhancement, supports chemotherapy, metabolic syndrome and diabetes support	[57,58,59,60]
Pestolan	*Pestolatia* sp.	Linear β-(1→3)	Antitumor activity, macrophage activation, experimental immunomodulator	[61,62,63]
Coriolan	*Coriolus versicolor*	Linear β-(1→3)	Anticancer immunotherapy, enhances T-cell and NK activity, adjunct in gastric and colorectal cancers	[42]

## Data Availability

No new data were created or analyzed in this study. Data sharing is not applicable to this article.

## Abbreviations

3-MA	3-Methyladenine
ASC	Apoptosis-Associated Speck-Like Protein Containing A CARD (Caspase Recruitment Domain)
ASD	Autism Spectrum Disorder
BALB/c	Bagg Albino Strain C
Bcl10	B-Cell Lymphoma/Leukemia
BDG	Serum β-1,3-Glucan
BDNF	Brain-Derived Neurotrophic Factor
BNCT	Boron Neutron Capture Therapy
BYG	Baker’s Yeast β-Glucan
CARD9	Caspase Recruitment Domain-Containing Protein 9
CAT	Catalase
CCL2	Chemokine (C-C motif) ligand 2
CCR2	C-C Chemokine Receptor Type 2
CD5	Cluster Of Differentiation 5
CG	Chitin–Glucan
CLP	Cecal Ligation And Puncture
COX-2	Cyclooxygenase-2
CQ	Chloroquine
CR3	Complement Receptor 3
CVD	Cardiovascular Disease
CXCL8	Chemokine (CXC Motif) Ligand 8
DNA	Deoxyribonucleic Acid
DSS	Dextran Sulfate Sodium
EphA2	Erythropoietin-Producing Hepatocellular
ERK MAP	Extracellular Signal-Regulated Kinase Mitogen-Activated Protein
FDA	Food And Drug Administration
GALT	Gut-Associated Lymphoid Tissue
GBM	Glioblastoma
GD2/GD3	Disialoganglioside GD2 / Disialoganglioside GD3
GEM	Gemcitabine
GLP-1	Glucagon-Like Peptide-1
GM-CSF	Granulocyte–Macrophage Colony-Stimulating Factor
GPX1	Glutathione Peroxidase
grifolan-LE	Grifolan (Lentinan-Like)—LE Variant
HDL	High-Density Lipoprotein
hemITAM	Hem Immunoreceptor Tyrosine-Based Activation Motif
HepG2	Human Hepatocellular Carcinoma Cell Line
IBD	Inflammatory Bowel Disease
IDO1	Indoleamine 2,3-Dioxygenase 1
IFIs	Fungal Infections
IFN-γ	Interferon-Gamma
IL	Interleukin
Imprime PGG	Imprime PGG (Poly-1,6-Β-D-Glucopyranosyl-Β-1,3-D-Glucopyranose)
iNOS	Inducible Nitric Oxide Synthase
IRE	Irreversible Electroporation
ITAM	Immunoreceptor Tyrosine-Based Activation Motif
JAM-1	Junctional Adhesion Molecule-1
LacCer	Lactosylceramide
LNT	*Lentinus Edodes* β-Glucan
LPS	Lipopolysaccharide
Malt1	Mucosa-Associated Lymphoid Tissue Lymphoma Translocation Protein 1
MAP	Mitogen-Activated Protein
MAPK–Elk-1	Mitogen-Activated Protein Kinase—Elk-1
MAPK–PPARγ	Mitogen-Activated Protein Kinase—Peroxisome Proliferator-Activated Receptor Gamma
MARCO	Macrophage Receptor With Collagenous Structure
MDA	Malondialdehyde
MDSCs	Myeloid-Derived Suppressor Cells
MHC-II	Major Histocompatibility Complex Class II
MIP-2	Macrophage Inflammatory Protein-2
MPO	Myeloperoxidase
mTOR	*Mechanistic (Or Mammalian) Target Of Rapamycin*
MyD88	Mal Adaptor Complex
NF-κB	Nuclear Factor Kappa-Light-Chain-Enhancer Of Activated B Cells
NFAT	Nuclear Factor Of Activated T Cells
NK cells	Natural Killer Cells
NLR	Neutrophil-To-Lymphocyte Ratio
NLRP3	NOD-, LRR- And Pyrin Domain-Containing Protein 3
NMR	Nuclear Magnetic Resonance
NO	Nitric Oxide
NSG mouse	NOD Scid Gamma Mouse
OVX	Ovariectomized
*p*-Akt	Phosphorylated Akt (Protein Kinase B)
PAMPs	Pathogen-Associated Molecular Patterns
PBMCs	Peripheral Blood Mononuclear Cells
PD-L1	Programmed Death-Ligand 1 (CD274)
PDAC	Pancreatic Ductal Adenocarcinoma
PEG2	Prostaglandin E2
PI3K	Phosphoinositide 3-Kinase
PKC	Protein Kinase C
PRRs	Pattern-Recognition Receptors
PSA	Prostate-Specific Antigen
RA	Rheumatoid Arthritis
RANTES	Regulated Upon Activation, Normal T Cell Expressed And Secreted
RAW264.7	Murine Macrophage Cell Line RAW 264.7
Rho GTPases	Ras Homolog Family Small Gtpases
ROS	Reactive Oxygen Species
RTK	Receptor Tyrosine Kinase
SCFAs	Short-Chain Fatty Acids
SDL	Superfine Dispersed Lentinan
SOD1	Superoxide Dismutase
SOX	A Standard Chemotherapy Regimen Consisting Of S-1 And Oxaliplatin
SR-A/CD204	Scavenger Receptor Class A / Cluster Of Differentiation 204
SRCD5CAR-NK	Shorthand For Chimeric Antigen Receptor (CAR) Natural Killer Cells Targeting SR-C/D5
SRs	Scavenger Receptors
STAT1	Signal Transducer And Activator Of Transcription 1
STAT3	Signal Transducer And Activator Of Transcription 3
Syk	Spleen Tyrosine Kinase
TGF-β	Transforming Growth Factor-β
TLRs	Toll-Like Receptors
TNBC	Triple-Negative Breast Cancer
TNF	Tumor Necrosis Factor
Tregs	Regulatory T Cells
Vav1	Vav Guanine Nucleotide Exchange Factor 1
VLDL	Very-Low-Density Lipoprotein
ZO	Zonula Occludens
